# Targeting the unfolded protein response for cancer therapy: mitigating tumor adaptation and immune suppression

**DOI:** 10.1186/s40364-025-00813-y

**Published:** 2025-12-11

**Authors:** Bilal Unal, Fahri Saatcioglu

**Affiliations:** 1 Department of Biosciences, University of Oslo, Postboks 1066, Blindern, 0316 Oslo, Norway; 2 Institute for Cancer Genetics and Informatics, Oslo University Hospital, Oslo, Norway; 3 Department of Molecular Oncology, Institute of Cancer Research, Oslo University Hospital, Oslo, Norway

**Keywords:** Cancer, Tumor microenvironment, Endoplasmic reticulum stress, Unfolded protein response, IRE1alpha, PERK, ATF6, Cancer immunotherapy, Immune check point inhibitors, PD-1

## Abstract

There are significant stress factors within the tumor microenvironment (TME), such as hypoxia, oxidative stress, and nutrient deprivation. These disrupt endoplasmic reticulum (ER) function in cancer cells, as well as the infiltrating immune cells, leading to activation of the unfolded protein response (UPR) signaling, which the tumor uses to mitigate stress and survive. There are three canonical UPR pathways that are regulated by respective ER-resident transmembrane sensors: inositol-requiring protein 1α (IRE1α), PKR-like ER kinase (PERK), and activating transcription factor 6 (ATF6); activation of these pathways results in expression of cognate transcription factors that regulate gene expression to mitigate ER stress. Persistent UPR activation in the TME has been linked to aberrant tumor growth, progression, metastasis, angiogenesis, and therapy resistance in different cancer types. In addition, modulation of UPR activity significantly impacts immune cell function at different levels further impacting its role on the TME. Therefore, there is now significant interest to design novel therapies that target the UPR to kill cancer cells and simultaneously enhance protective anti-tumor immunity. Here we summarize recent findings as to how targeting UPR signaling can induce tumor regression and at the same time galvanize the immune response. We discuss the potential of integrating UPR targeting with other therapies, such as immune checkpoint inhibition, highlighting emerging strategies to improve therapeutic efficacy and overcome resistance. These recent insights underscore the importance of UPR as a novel therapeutic target for cancer treatment.

## Background

Endoplasmic reticulum (ER) is the largest organelle in eukaryotic cells. It is composed of a continuous lipid bilayer that is linked to the outer nuclear membrane and extends throughout the cytoplasm making physical and functional contacts with all other organelles. The synthesis, folding, and transport of proteins that are destined for the endomembrane system and exocytosis, comprising about one third of the proteome, takes place in the ER; thus, ER is indispensable for the maintenance of normal cellular physiology and homeostasis. ER is also the major storage site for cellular calcium and has key roles in lipid synthesis and carbohydrate metabolism [1]. These functions are mediated by the two types of ER that are categorized based on their structural appearance under the electron microscope [1, 2]: The rough ER is studded with ribosomes and is responsible for protein synthesis, whereas smooth ER is devoid of ribosomes and instead has important roles in calcium homeostasis, steroid hormone production and lipid biosynthesis [1, 3, 4].

Upon recognition of cytosolic mRNA transcripts by membrane bound ribosomes, translation of proteins destined for cellular membranes or secretion occurs through nascent peptide synthesis that protrude into the ER lumen. Once in the ER, folding and post-translational modifications take place, such as disulfide bond formation and glycosylation with the help of chaperones and other protein modifying enzymes. Subsequently, proteins are packed into vesicles for transport to cytosolic membranes, the plasma membrane, or for secretion outside of the cell [1, 5, 6].

Maintaining protein folding fidelity in the ER lumen is essential for normal cellular function. Various stress factors such as hypoxia, electrolyte imbalance, and oncogene activation, or loss of tumor suppressor gene expression, can trigger accumulation of misfolded or unfolded proteins in the ER, called ER stress, that is detrimental to the cell and if unchecked can result in cell death [7, 8]. Therefore, in response to ER stress, a comprehensive adaptive mechanism is initiated, termed the unfolded protein response (UPR), which reprograms the cellular transcriptional, translational, and degradation pathways to alleviate ER stress [9, 10].

## Three arms of the unfolded protein response

**Fig. 1:**
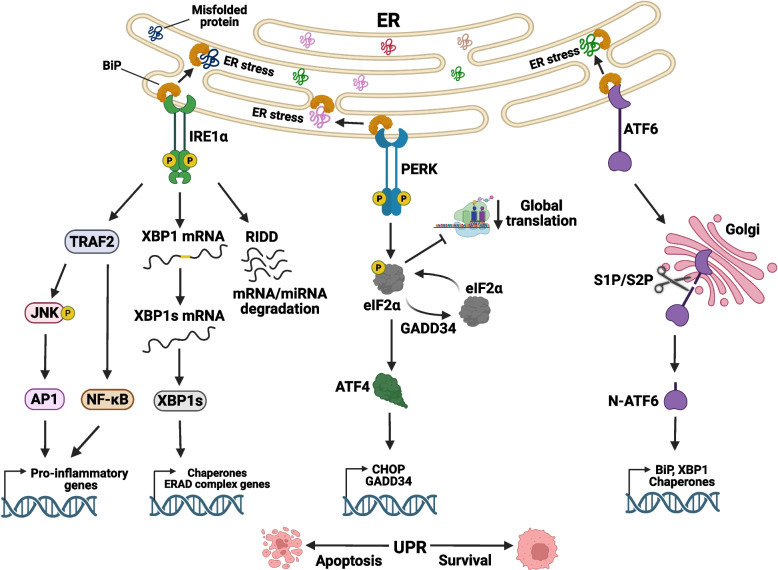
The unfolded protein response (UPR) stress signaling pathways. The three canonical UPR arms are schematically shown. In unstressed cells, the three UPR sensors, IRE1α, PERK, and ATF6 kept inactive by binding to BiP or by direct binding to unfolded proteins. Upon stress and an increase in unfolded proteins in the ER lumen, BiP dissociates from the ER stress sensors to aid in protein folding; this results in activation of the three UPR arms that ultimately activate transcriptional regulators XBP1s, ATF4, and ATF6; these in turn activate gene expression programs to facilitate protein folding, modulate protein synthesis, degradation and secretion to establish cellular homeostasis. When activated, IRE1α and/or PERK oligomerize and trans-autophosphorylate (P) themselves. Active IRE1α through its unconventional RNase activity removes an intron in the *XBP1* mRNA, resulting in the mRNA for *XBP1s*, which encodes the active transcription factor XBP1s that translocates to the nucleus and activates UPR target gene expression. In the case of severe ER stress, IRE1α initiates non-selective mRNA or miRNA degradation through RIDD and through TRAF2 recruitment, which activates JNK and NF-κB signaling to drive the expression of pro-inflammatory cytokines. Active PERK phosphorylates eIF2α, which inhibits general translation initiation but promotes translation of specific transcripts such as ATF4 and CHOP. ATF4 then translocates to the nucleus and activates its target genes, including CHOP, which can trigger apoptosis. Another target of ATF4, the phosphatase GADD34, reverses eIF2α phosphorylation that re-initiates protein translation. Upon activation, ATF6 translocates to the Golgi apparatus, cleaved by S1P and S2P site specific proteases, resulting in the release of active cleaved ATF6 (N-ATF6) transcription factor that translocates into the nucleus to activate UPR target gene expression

UPR is mediated by the activation of three evolutionarily conserved ER-membrane resident mediators: inositol-requiring protein 1α (IRE1α), PKR-like ER kinase (PERK), and activating transcription factor 6 (ATF6) (Fig. 1). These sensors are kept inactive by the chaperone binding immunoglobulin protein (BiP, also known as 78 kDa glucose-regulated protein, GRP-78) in unstressed cells [9, 10]. Upon ER stress, BiP dissociates from the sensors to bind to and increase the folding capacity of proteins in the ER lumen. Once free of BiP, the three ER stress sensors initiate signaling events resulting in the expression/maturation of transcription factors that regulate gene expression; the protein products of these are involved in reversing ER stress and re-establishing cellular homeostasis **(**Fig. 1**)** [11, 12]. If chronic ER stress remains unresolved, the same UPR pathways activate cell death pathways. Thus, UPR is a two-edged sword and must be tightly regulated for maintenance of normal cell function and physiology [13, 14]. Given this central position in normal cells, it is not surprising that UPR pathways are dysregulated in a number of human diseases, such as cancer, neurodegenerative diseases, and metabolic disorders, which we come back to below after describing the three canonical arms in more depth [11, 12].

### IRE1α arm

IRE1α (encoded by endoplasmic reticulum to nucleus signaling 1 gene, ERN1) is the evolutionarily most conserved UPR sensor that was originally identified in yeast [15]. IRE1α is ubiquitously expressed, whereas the expression of IRE1 beta (IRE1β), encoded by another gene that is a homolog of IRE1α, is predominantly limited to the digestive system and the epithelial lining of the respiratory tract [16, 17]. Although less well studied, IRE1β has some important functions: it promotes goblet cell maturation, regulates mucin 2 secretion, and maintains the intestinal barrier. Its loss disrupts mucus layer integrity, impairs host–microbiota interactions, and increases susceptibility to inflammation-driven and genetic tumorigenesis [18–22].

During ER stress, misfolded proteins induce the release of chaperones, such as BiP, from IRE1α; alternatively, misfolded proteins directly bind the luminal N-terminal domain of IRE1α. This activates the serine-threonine kinase and/or the atypical sequence specific endoribonuclease (RNase) domain of IRE1α in its cytosolic C-terminal domain [23]. IRE1α activation results in its oligomerization and trans-autophosphorylation that induces selective triggering of its RNase domain which cleaves a 26 base-pair segment from the transcription factor X-box binding protein 1 (*XBP1*) mRNA. The resulting spliced mRNA is then joined by RNA 2',3'-cyclic phosphate and 5'-OH ligase (RTCB) that catalyzes ligation of the 5’-OH and 2’,3’-cyclic phosphate of the two RNA fragments to form spliced *XBP1* (*XBP1s*) mRNA, which is translated into the active transcription factor XBP1s [24–27]. XBP1s then translocates into the nucleus and activates target gene expression, such as for chaperones and genes involved in ER-associated degradation (ERAD) to alleviate ER stress [28] (Fig. 1). If the ER stress is severe, this leads to hyperphosphorylation and homo-oligomerization of IRE1α that results in preferential non-selective degradation of ER-localized mRNAs through a process called regulated IRE1-dependent decay (RIDD) [29]. Targets of RIDD also include micro-RNAs (miRNAs) (e.g. miR-17, miR-34a, miR-96, miR-125), and mRNAs in different cellular locations (e.g. *BLOC1*, *CD59*, *GALNT2*, *COL6A1*, *MKRN2*) [29]. Some miRNAs that inhibit the expression of pro-apoptotic genes, such as apoptosis signal-regulating kinase 1 (ASK1*)*, can also be targeted by the RIDD machinery [30]. For example, miRNA-301a suppresses *ASK1* expression and c-Jun N-terminal Kinase (JNK) activation during hypoxia in human adipose-derived stem cells and it is a target of IRE1α RIDD activity [31]. miRNA-19a and miR-199a-5p, also RIDD targets, similarly regulate *ASK1* expression in endothelial cells and in smooth muscle cells, respectively [32, 33], whereas miRNA-301a, suppresses *ASK1* expression and activation of JNK during hypoxia in human adipose-derived stem cells [31]. miRNA-19a and miR-199a-5p were also reported to regulate *ASK1* expression in endothelial cells and in smooth muscle cells, respectively [32, 33]. These data establish RIDD activity of IRE1α as a major determinant in life/death decisions in a variety of cells.

In addition to its well-characterized functions in XBP1 splicing and RIDD-mediated RNA degradation, recent studies have uncovered broader roles of IRE1α in regulating key oncogenic signaling pathways and microRNA networks. Beyond its ability to activate ASK1-JNK signaling through its RIDD activity, IRE1α promotes PI3K/AKT signaling via the XBP1 axis during ER stress. In melanoma cells, XBP1 was shown to upregulate AKT phosphorylation, contributing to resistance to chemotherapeutic agents such as docetaxel and vincristine [34]. More recently, Liu et al. demonstrated that the small molecule verteporfin acts as a molecular glue, inducing IRE1α dimerization and activation, which leads to XBP1 splicing and miR-153-mediated suppression of PTEN, thereby enhancing AKT phosphorylation in breast cancer (BCa) models [35]. This reveals a mechanistic link between IRE1α-XBP1 signaling and the PI3K/AKT survival pathway. Furthermore, while IRE1α promotes miRNA degradation via RIDD, emerging evidence suggests that it can also induce miRNA expression through XBP1s-mediated transcriptional regulation. For instance, in acute myeloid leukemia (AML), XBP1s was shown to transcriptionally upregulate miR-22-3p by directly targeting its host gene, MIR22HG, leading to downregulation of SIRT1 and increased chemosensitivity [36]. Together, these findings highlight the dual role of IRE1α in both degrading and inducing specific miRNAs, and its broader influence on survival pathways such as AKT, expanding its relevance as a therapeutic target in cancer.

IRE1α phosphorylation also leads to the recruitment of tumor necrosis factor receptor-associated factor 2 (TRAF2) and ASK1 to the cytosolic domain of IRE1α resulting in their activation [13, 37] (Fig. 1). This results in the activation of key inflammatory signaling pathways such as JNK and Nuclear factor kappa-light-chain-enhancer of activated B cells (NF-κB). IRE1α and TRAF2 forms a complex that triggers the activation of inhibitor of NF-kB alpha (IκBα) kinase (IKK) that leads to IκBα phosphorylation and its proteasomal degradation; this results in the release of NF-κB, which translocates to the nucleus and orchestrates the expression of many inflammatory response genes [38]. In addition, IRE1α-TRAF2-mediated JNK activation leads to phosphorylation of Activator protein 1 (AP-1), which can also initiate the transcription of inflammatory gene programs as well as apoptosis. Thus, ER stress not only triggers the activation of UPR through IRE1α, but during prolonged stress conditions, it can also activate the inflammatory gene regulatory programs and is also involved in cell death signaling [39].

### PERK arm

Eukaryotic translation initiation factor 2 alpha kinase 3 (EIF2AK3 or PERK) is a protein kinase which is activated through dimerization and trans-autophosphorylation during ER stress upon dissociation from BiP [40]. PERK then phosphorylates the alpha subunit of eukaryotic initiation factor-2 (eIF2α) at serine 51, which in turn leads to competition of p-eIF2α with eIF2B and reduced rate of ternary complex formation, resulting in inhibition of general protein translation initiation; this leads to reduction in the number of proteins in the ER and helps relieve ER stress [9, 10, 41, 42] (Fig. 1). During this process, some transcripts, such as those for activating transcription factor 4 (*ATF4*) and CCAAT/enhancer binding protein (C/EBP) homologous protein (*CHOP*), are selectively translated to regulate the stress response. This is mediated via upstream open reading frames (uORF) that influence the translation of downstream main coding sequences (CDS) within these mRNA molecules [43, 44]. Under normal physiological conditions, where the ternary complex is abundant, ribosomes initiate scanning at the uORF1 of the ATF4 transcript, swiftly reinitiating at uORF2. However, uORF2 is out-of-frame with the ATF4 CDS that aborts ATF4 translation. Under ER stress conditions, the limited availability of the ternary complex triggers extended ribosomal scanning across the ATF4 transcript, facilitating translation initiation at the ATF4 CDS [45]. Once produced, ATF4 translocates to the nucleus and regulates gene expression, such as increasing the expression of chaperone genes, including crosstalk with the IRE1α-XBP1s pathway, to decrease the incoming stress [9, 10, 46].

PERK was recently reported to attenuate IRE1α activity through the phosphatase RNA polymerase II associated protein 2 (RPAP2), which reverses IRE1α phosphorylation, oligomerization, and activation [47]. This inhibits IRE1α dependent adaptive processes, including the activation of cytoprotective factors and ERAD [47]. Moreover, ATF4 increases expression of genes involved in apoptosis such as *CHOP*, inhibits expression of anti-apoptotic genes such as *BCL2*, induces negative feedback regulation by eIF2α dephosphorylation (through DNA damage inducible and growth arrest protein, GADD34), and induces calcium release to trigger cell death [48–50]. PERK-induced CHOP expression upregulates GADD34 production that orchestrates the recruitment of serine/threonine protein phosphatase 1 (PP1) to dephosphorylate p-eIF2α. This process restores protein synthesis levels when ER stress has been resolved [47, 51].

**Fig. 2:**
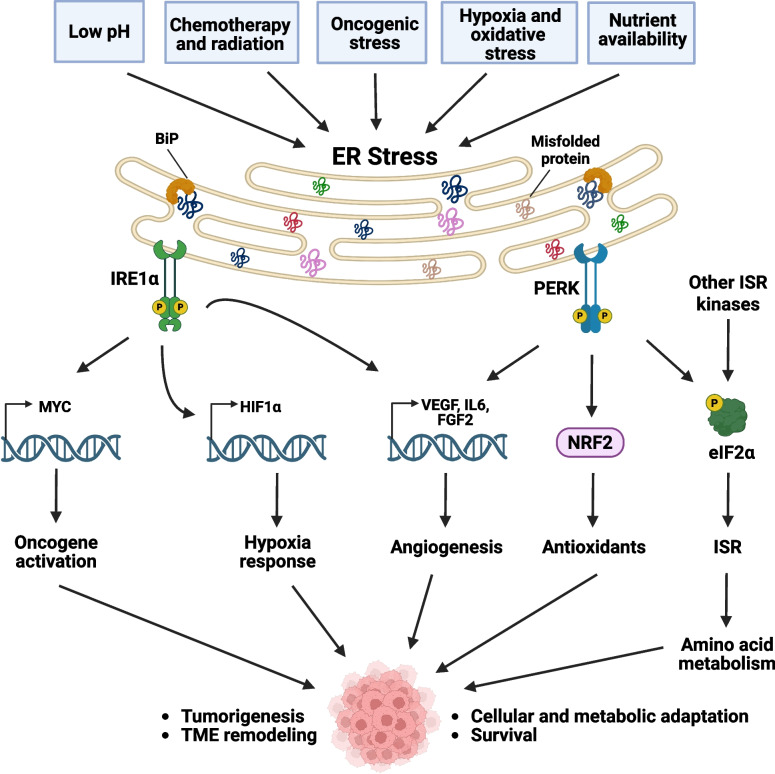
Activation of UPR pathways rewires cancer cell metabolism, drives cellular adaptation, and remodels the TME to promote survival and tumorigenesis. Tumor cells are constantly exposed to stressors (oncogene activation, hypoxia, nutrient deprivation, misfolded proteins, oxidative stress, acidosis, chemotherapy, and radiation) in the TME, leading to activation of UPR, mainly IRE1α and PERK arms, but also ATF6. Precancerous cells undergo UPR activation in response to oncogenic stress. Various genetic and epigenetic changes, particularly affecting oncogenes or tumor suppressor genes, drive tumor development. Activation of oncogenes such as RET or RAS and the loss of tumor suppressors such as PTEN, lead to increased protein synthesis due to increased metabolic demands during malignant transformation. This activates the UPR via PERK and IRE1α pathways. The interaction of IRE1α and PERK pathways can promote cell survival by fostering tumorigenesis, or initiate protective responses, such as apoptosis and senescence, to prevent tumorigenesis. External factors in the TME can trigger UPR activation in cancer cells. Environmental factors, such as amino acid scarcity, oxidative stress, hypoxia, and deficiency in protein N-glycosylation (glucose deficiency reducing the hexosamine pathway) significantly promote UPR activation. Oxygen and glucose deprivation induce protein misfolding and activate the UPR, primarily through the PERK and IRE1α pathways. These pathways foster cell survival and angiogenesis. Furthermore, cancer cells can ameliorate oxidative stress through the PERK-NRF2 pathway and glutathione biosynthesis. Additionally, in PCa, XBP1s regulates MYC oncogene expression via IRE1α-XBP1s signaling, promoting tumorigenesis and survival. Lastly, hypoxia, oxidative stress, and nutrient deficiency activate PERK and/or other kinases not involved in the UPR but that are part of the integrated stress response (ISR). These kinases phosphorylate eukaryotic initiation factor 2α (eIF2α) and trigger signaling pathways to adjust amino acid metabolism and redox balance

One of the important consequences of ER stress is an increase in oxidative stress through accumulation of reactive oxygen species (ROS). For this reason, the UPR is also integrated with the antioxidant protection system: PERK plays an essential role by activating the nuclear factor erythroid 2-related factor 2 (NRF2) pathway through dissociation of the NRF2/Keap1 complex, resulting in translocation of the transcription factor NRF2 to the nucleus (Fig. 2) [52]. NRF2 and ATF4 then orchestrate the expression of antioxidant genes such as glutathione S-transferases (GSTM1 and GSTP1), thioredoxin reductase 1 (TXNRD1), and hemeoxygenase-1 (HO-1) [53], to engage in survival responses. Thus, PERK is not only involved in UPR regulation, but also has an important role to mitigate the oxidative stress that is increased during ER stress.

In addition, IRE1α and PERK have been demonstrated to have cellular roles that extend beyond their enzymatic functions, operating via mechanisms involving interactions with other proteins [54–57]. For example, Mitochondria-associated membranes (MAMs) orchestrate bioenergetics through calcium transfer from the ER to mitochondria. Independent of its enzymatic activity, IRE1α can act as a structural determinant within MAMs by interacting with inositol-1,4,5-trisphosphate (InsP3) receptors (InsP_3_Rs) to regulate mitochondrial calcium uptake [58]. Moreover, in multiple myeloma (MM), IRE1α regulates phosphorylation of Interferon regulatory factor 4 (IRF4), a key transcription factor for tumor cell proliferation. IRE1α silencing increases inhibitory IRF4 phosphorylation, reducing its activity and slowing tumor growth. Restoring IRF4 reverses this effect, highlighting IRE1α’s role beyond RNA splicing [59]. In addition, some cancer cells rely on IRE1α independently of its enzymatic activity or XBP1 splicing. IRE1α knockdown, but not inhibition of its enzymatic function, impaired cell cycle progression and tumor growth by activating p53/p21 and increasing DNA damage, emphasizing a critical non-enzymatic function of IRE1α in cancer biology [60]. Furthermore, PERK facilitates lipid movement at the junction between the ER–mitochondria contact sites (EMCs) through an unconventional mechanism that is independent of UPR. As an adaptor, PERK recruits the lipid transfer protein Extended-Synaptotagmin 1 (E-Syt1) to EMCS. This interaction facilitates phospholipid transfer between the ER and mitochondria in resting cells [61]. Many other protein–protein interactions for IRE1α and PERK have been identified with emerging potential significance [54–57].

### ATF6 arm

The activating transcription factor 6 alpha (ATF6α, referred to as ATF6 hereafter) is a basic leucine zipper (bZIP) transcription factor that is tethered to the ER membrane in resting cells [62]. ATF6 beta (ATF6β) is encoded by an independent gene that poorly activates UPR target genes due to the absence of eight critical amino acids within the transcriptional activation domain (TAD) and thus its exact function is poorly understood [62]. In contrast to IRE1α and PERK, during ER stress, BiP dissociates from ATF6, exposing Golgi localization sequences. This allows ATF6 to translocate to the Golgi apparatus, where it is cleaved by the Golgi-resident proteases Site-1 (S1P) and Site-2 (S2P) (Fig. 1) [63, 64]. This liberates the N-terminal domain of ATF6, which is active transcription factor, which then translocates into the nucleus and promotes the expression of UPR target genes, including those encoding molecular chaperones, proteins responsible for ER expansion, proteins that degrade misfolded proteins, and ERAD [9, 10, 65].

A disulfide bond in monomeric ATF6 is required for its release from ER, trafficking to the Golgi, and subsequent proteolysis [65]. Upon ER stress and BiP dissociation, ATF6 undergoes a redox switch, forming a disulfide-bonded dimer that then translocates to the Golgi and is cleaved by the SP1 protease [66]. Thioredoxin domain containing 12 (ERp18), an ER-resident oxidoreductase, is a key orchestrator in this process, influencing ATF6 dimerization, Golgi trafficking and activation during stress [66, 67]. Protein disulfide isomerase family A member 5 (PDIA5) was also found to orchestrate disulfide bond rearrangement in ATF6, prompting its export from the ER and subsequent target gene activation [68].

One of the target genes of ATF6 is XBP1; as reviewed above, under stress conditions XBP1 is cleaved by IRE1α to generate XBP1s; thus ATF6 directly contributes to the activation of the IRE1α arm of UPR. Furthermore, ATF6 upregulates the ER stress induced protein P58IPK (DNAJC3) expression, which is a negative modulator of eIF2α activity, thereby also providing input to PERK signaling [69, 70]. Thus, there is a significant crosstalk between ATF6 and other two canonical UPR arms, which concurrently orchestrate the UPR and cell death pathways upon accumulation of misfolded proteins in the ER lumen.

In summary, ER is a functionally indispensable organelle with complex mechanisms to maintain protein homeostasis, making important contacts with all parts of the cell; it is also the site for UPR that is essential for life/death decisions under ER stress conditions. Recent evidence has suggested that UPR signaling is frequently hijacked by cancer cells, wherein they are used for survival and escape from anti-tumor immunity to promote tumorigenesis under hostile conditions, such as hypoxia and limited nutrient availability [71–74]. Thus, targeting UPR pathways is an emerging key area in cancer therapeutics, as well as in a number of other pathological conditions, such as neurodegenerative diseases. Below, we review the recent findings in this fast-advancing field as it relates to cancer.

### UPR and cancer

Cancer cells are constantly exposed to a myriad of stressors, including hypoxia, nutrient deprivation, acidosis, oxidative stress from mitochondrial dysfunction, and increased demands for protein synthesis due to rapid proliferation **(**Fig. 2**)**. During therapy, cancer cells face additional challenges from chemotherapy and radiation [75, 76], necessitating robust adaptive mechanisms to ensure their survival. The UPR has recently emerged as a critical pathway that cancer cells often hijack to cope with these stresses [71–74]. Its precise regulation is essential, since UPR can determine the fate of cancer cells, whether they will survive or undergo apoptosis, thus significantly influencing tumor development and progression.

Activation of UPR signaling has been implicated in a number of cancer types, including prostate cancer (PCa), breast cancer (BCa), multiple myeloma, pancreatic cancer, non-small cell lung cancer, colorectal cancer, and hepatocellular carcinoma [72, 77–79]. For example, recent studies indicate that steroid hormones, such as androgens in PCa and estrogens and progesterone in BCa, activate UPR pathways in both normal and malignant cells. Since antihormone therapies are mainstay treatments in the clinic for both PCa and BCa, targeting the UPR could be a novel therapeutic strategy for hormone-regulated cancers (for a review, see [72]).

A number of the molecular mechanisms of UPR and cancer promotion has already been identified. For example, IRE1α-XBP1s signaling is a key regulator of MYC mRNA and protein expression, driving tumorigenesis and cellular survival [80]. Consistently, pharmacological targeting of IRE1α RNase function with the small molecule inhibitor MKC8866 significantly reduced tumor growth in preclinical PCa models [81, 82]. In BCa, MYC regulates IRE1α transcription, forming a transcriptional complex with XBP1s that helps alleviate MYC-induced proteotoxic stress and restore ER homeostasis. XBP1s has emerged as a synthetic lethal partner of MYC; silencing XBP1s selectively inhibits growth in MYC-driven BCa, further reinforcing the potential of targeting the IRE1α/XBP1 pathway in therapeutic strategies [83]. Notably, MKC8866 inhibited tumor growth in MYC-overexpressing BCa patient derived xenograft (PDX) models and enhanced the efficacy of docetaxel [83]; in addition, in pancreatic cancer, MKC8866 treatment helped overcome resistance to KRAS inhibitors leading to rapid tumor regression [84], indicating its potential therapeutic promise in multiple cancer types.

In addition to activating proliferative pathways, the hijacking of UPR pathways allows cancer cells to also evade cell death. For instance, XBP1s promotes the transcription of pro-survival genes in in PCa; consistently, genetic targeting genetic targeting of IRE1α or XBP1s results in reduced tumor growth and increased sensitivity to apoptosis [85, 86]. In addition, PC3 PCa cells exposed to the bioactive lipid mediator sphingosine 1-phosphate exhibited extrinsic ER stress, which induced autophagy through increased XBP1s splicing [87]. Activation of ATF6 can also protect cancer cells from ferroptosis, a form of regulated cell death linked to iron accumulation and lipid peroxidation [88]. The ATF6 small molecule inhibitor Ceapin-A7 has synergistic effects with anti-androgens, such as enzalutamide, in PCa models [88], further underscoring the therapeutic potential of targeting UPR pathways.

UPR activation also facilitates cancer cell metastasis in a number of cancer types, such as BCa, colorectal cancer, and oral and esophageal squamous cell carcinoma [89, 90]. Both IRE1α-XBP1s and PERK-ATF4 pathways are upregulated in various primary as well as metastatic cancers, by facilitating epithelial-to-mesenchymal transition (EMT) and extracellular matrix (ECM) remodeling [89, 90]. For example, ectopic expression of ATF4 in esophageal squamous cell carcinoma has been linked to increased migration and invasion, mediated by the expression of matrix metalloproteinases (MMPs) [91]. In PCa, the molecular chaperone HSP27 plays a significant role in facilitating cell invasion and migration through the regulation of MMP2 expression [92].

Additionally, UPR pathways contribute to tumor cell dormancy, a phenomenon where cancer cells enter a quiescent state, often arrested at the G0–G1 phase of the cell cycle, until conditions in the TME become favorable for proliferation [83–85]. The PERK signaling has been associated with G0–G1 cell-cycle arrest, suggesting that UPR activation is vital for cancer cells to endure adverse conditions during metastatic spread and dormancy [93, 94]. Moreover, targeting UPR pathways has been shown to sensitize chemoresistant cells to conventional therapies [90], emphasizing their importance in developing more effective, combinatorial treatment strategies. For example, in BCa, combination of XBP1 short hairpin RNA with doxorubicin can significantly inhibit tumor growth in xenograft models, highlighting the role of IRE1α-XBP1s signaling in mediating chemoresistance [95]. Consistently, blocking IRE1α by MKC8866 in PCa, BCa, and pancreatic cancer mouse models led to synergistic effects with the clinically approved anti-cancer drugs, such as enzalutamide, abiraterone, cabazitaxel, docetaxel, sotarasib, and trametinib, all of which are known to eventually lose efficacy in the clinic due to development of resistance [81, 83, 84, 96]. However, it remains unclear as to whether targeting the UPR could resensitize chemoresistant cells and lead to more effective therapy responses in patients.

In summary, the UPR is a vital player in cancer cell survival, tumorigenesis, metastasis, and resistance to therapy. The multifaceted roles of UPR underscore its potential as a therapeutic target, with ongoing research aimed at developing strategies that can effectively disrupt UPR signaling in cancer cells, either alone or with existing therapies.

## UPR and the tumor microenvironment

### Factors fostering ER stress in the TME

During tumor development, cancer cell growth mainly depends on their ability to adapt to the stress factors in the TME such as low pH, limited oxygen, oxidative stress, oncogenic stress, and deficient nutrient supply **(**Fig. 2**)**. These conditions in the TME not only disrupt the ER folding capacity of cancer cells but also significantly disturb the immune cells that weaken their function and promote the ability of cancer cells to evade host immunity [79, 97]. The occurrence of oncogenic events in cancer cells such as loss of tumor suppressors (p53, TSC1, TSC2, and PTEN) [98–100] and activation or overexpression of oncogenic pathways (HRAS, BRAF, and RET) [95, 101, 102] exacerbates ER stress by increasing the overall rates of protein translation which result in nutrient deprivation, acidosis and hypoxia, as well as challenging the protein folding capacity leading to ER stress [71, 95, 103, 104] (Fig. 2). As in cancer cells, following accumulation of misfolded or unfolded proteins, the UPR is triggered as a mechanism to restore homeostasis and facilitate adaptation to various challenges within other cells in the TME [9]. In addition, some cancer therapeutics, such as chemotherapy and radiation, can trigger ER stress in cancer cells as well as the immune cells in the TME [105]. The response to ER stress in the TME can vary depending on the severity of the stress, cell type, and the specific context of the malignancy. These responses can encompass a range of effects, including cellular reprogramming, adaptation, autophagy, and apoptosis [48, 106–109]. In summary, several internal and external factors contribute to the activation of ER stress not only in cancer cells, but also in immune cells within the TME with important functional outcomes. Below we present a summary of current findings regarding activation of ER stress through microenvironmental factors.

### Hypoxia and oxidative stress

Lack of oxygen in the TME disrupts the equilibrium within the ER and induces cellular stress [110–112] (Fig. 2). Reduced oxygen levels also activate complex III of the mitochondrial electron transport chain, leading to increased production of ROS in the cytosol that are necessary for stabilizing the hypoxia-inducible transcription factor alpha (HIF1α) [113]. In addition, ROS can produce peroxidized lipid byproducts that are highly reactive and can form damaging covalent adducts with various ER chaperones [114, 115]. Furthermore, the signaling processes triggered by the detection of pro-inflammatory cytokines, growth factors, or the engagement of pattern recognition receptors (PRRs) can lead to continuous activation of downstream NADPH oxidases (NOXs) that results in the production of large quantities of ROS [116], which can then lead to severe ER stress and UPR activation [117]. For example, IRE1α and PERK arms of UPR have been implicated in hypoxia resistance and tumor growth [118–120]. Mouse embryonic fibroblasts (MEFs) deficient for XBP1 or PERK resulted in tumor growth inhibition due to augmented sensitivity to hypoxia when transplanted into mice [104, 121, 122]. Similarly, in triple-negative breast cancer (TNBC), the IRE1α-XBP1s pathway plays a critical role in remodeling cancer metabolism [95]. XBP1s physically interacts with HIF1α, a key regulator of the hypoxia response, to regulate the hypoxia response and glycolysis [95]. This interaction leads to expression of key metabolic regulators such as glucose transporter 1 (GLUT1) and lactate dehydrogenase A (LDHA) in TNBC [95].

Moreover, cancer cells can trigger PERK activation as an adaptive mechanism, which in turn stabilizes the nuclear factor erythroid 2-related factor 2 (NRF2) to mitigate oxidative damage by inducing glutathione synthesis [123]. While certain processes involved in protein synthesis can occur without oxygen, post-translational folding and isomerization, and lipid desaturation require molecular oxygen. Post-translational disulfide bond formation is oxygen dependent and disruption of this process contributes to hypoxia-induced ER stress [124]. In addition, hypoxia impairs the function of ER Oxidoreductase 1 alpha (ERO1α), an oxygen-dependent enzyme located in the ER that is essential for disulfide bond formation and protein folding [125]. Another consequence of hypoxia is the reduction in desaturated lipids, which in turn hinders ER expansion that can induce ER stress [126]. In summary, UPR signaling has major roles for the tumors to survive under hypoxia and oxidative stress.

### Nutrient availability

ER homeostasis can be disrupted by metabolic stress, which occurs when there is an imbalance between the nutrient supply and energy requirements of the cell (Fig. 2). This imbalance can be caused by both insufficient and excessive nutrient availability [73, 127]. ER stress can be influenced by the availability of glucose and glutamine in several ways. When there is a shortage of glucose or glutamine, this disrupts the hexosamine biosynthetic pathway (HBP), which relies on nutrients to produce uridine diphosphate-N-acetylglucosamine (UDP-GlcNAc) that is crucial for the process of N-linked glycosylation and proper protein folding within the ER [128, 129]. Moreover, glucose restriction affects ATP production which serves as an energy resource and phosphate donor for protein folding in the ER [130]. Insufficient glucose can also lead to disrupted calcium flux in the ER that is regulated by the activity of sarcoplasmic/ER calcium ATPase (SERCA) [131].

Inadequate availability of amino acids is another significant stress factor in the TME; when amino acids are scarce, activation of General control nonderepressible 2 (GCN2) kinase leads to phosphorylation of eIF2α that triggers the integrated stress response (ISR). The ISR has been recognized as a crucial mechanism for stress adaptation in cancer cells under amino acid deprivation [45, 132]. In a recent study, it was reported that ER stress and nutrient deprivation stimulated mitochondrial bioenergetics through PERK, promoting the formation of respiratory supercomplexes and enhanced mitochondrial respiration [133]. This reveals an important link between ER and mitochondria in cellular energy regulation under nutrient deficiency and ER stress.

### Low pH

Most cancer cells predominantly rely on aerobic glycolysis as their main metabolic pathway, leading to the production of lactic acid, which subsequently decreases the pH in the TME [134]. The activation of proton-sensing receptors in response to low pH can initiate the activation of UPR in different cell types [135, 136] (Fig. 2), which is believed to occur through disturbances in intracellular calcium levels and/or ROS generation [136–139].

### Chemotherapy and radiation

Development of resistance to chemotherapy is a major issue in cancer treatment in the clinic. Various modes of this resistance have been identified, including drug inactivation, efflux of the drug from the cell, increase in DNA damage repair, and induction of autophagy (for a review, [140]). Each of these mechanisms can be significantly mediated by UPR. For example, certain types of chemotherapies can cause severe ER stress responses that can lead to immunogenic cell death, resulting in protective anti-tumor immunity [105, 141–144]. One example is anthracycline family of cytotoxic drugs, which induce excessive production of ROS in cancer cells, leading to severe ER stress [105, 141–144] (Fig. 2). This results in the translocation of ER-associated chaperone calreticulin to the surface of cancer cells and release of damage-associated molecules (DAMPs), including double stranded DNA and high mobility group protein B1 (HMGB1). Calreticulin interacts with scavenger receptors and CD91, while HMGB1 activates Toll-like receptor 2 (TLR2) and/or TLR4 signaling on nearby phagocytic immune cells. These interactions initiate diverse signaling pathways that promote activation of dendritic cells (DCs) which are responsible for priming or reactivating tumor-specific T cells for protective anti-tumor immune responses [105, 141–144]. Recently, it was reported that the activating receptor NKp46 expressed on NK cells and some innate lymphoid cells recognize externalized calreticulin (ecto-CRT) which is translocated from the ER to the cell membrane during ER stress [145], which occurs during chemotherapy-induced immunogenic cell death [146]. The recognition of ecto-CRT by NKp46 triggers activation of NK cell signaling, leading to elimination of ER-stressed cells, inhibition of tumor growth, and enhanced immune responses in the TME [145].

Another mechanism of action of anthracyclines and certain pharmacological agents is their ability to induce tetraploidization in cancer cells specifically by increasing eIF2α phosphorylation. Interestingly, these treatments do not activate other factors involved in the ER stress response such as ATF4, ATF6, or XBP1s [147]. In contrast, IRE1α-XBP1s signaling was necessary for resistance to immunogenic cell death in colorectal cancer cells induced by combinatorial treatment of chemotherapy and cetuximab, a monoclonal antibody that blocks epidermal growth factor receptor (EGFR)-ligand interaction [148].

Radiation also has a major impact on UPR activation. Exposure to radiation results in damage to macromolecules, both directly or indirectly through the generation of ROS or reactive nitrogen species (RNS) [149]. This damage leads to oxidative stress which can disrupt the cellular homeostasis in the cell by DNA damage, mitochondrial dysfunction, and protein misfolding resulting in ER stress [150–152]. Additionally, ROS generation under radiation treatment triggers ER stress and activates the UPR [153] (Fig. 2). In a recent paper, it was reported that conformal radiotherapy promotes recruitment of interferon I-producing monocytes which potentiate anti-tumor immune responses in murine cancer models [154]. However, non-conformal radiotherapy was shown to cause damage to healthy tissues and led to reduction in the recruitment of monocytes into tumors, resulting in formation of an immunosuppressive TME [154].

## ER stress-induced immunosuppression within the TME

**Fig. 3:**
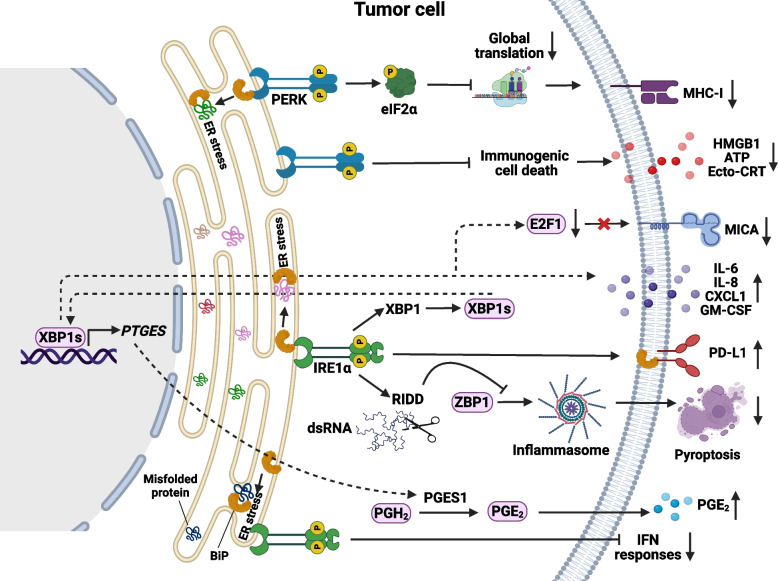
ER stress-mediated immunosuppression within the TME. Stress conditions in the TME, such as low pH, oxygen deprivation, oxidative stress, oncogenic signaling, and limited nutrient availability, impair the ability of ER to properly fold proteins. This leads to the accumulation of misfolded proteins and activates ER stress sensors in cancer cells, including IRE1α, PERK, and ATF6 (not shown), which influence anti-tumor immune responses. Top panel: PERK activation leads to phosphorylation of eIF2α, resulting in reduced global protein translation and downregulation of MHC-I expression that causes immune evasion of cancer cells. In melanoma, PERK suppresses immunogenic cell death, thereby limiting anti-tumor immunity. Bottom panel: XBP1s suppresses NK cell recognition of tumor cells by downregulating MICA expression through suppression of the transcription factor E2F1. In melanoma, IRE1α signaling enhances PD-L1 expression, while in TNBC, ER stress stabilizes PD-L1 via interaction with the chaperone BiP. In TNBC, the IRE1α–XBP1s pathway promotes the expression of cytokines such as IL-6, IL-8, CXCL1, and GM-CSF, facilitating the accumulation of CAFs and MDSCs, thereby weakening anti-tumor immunity. Through RIDD, IRE1α prevents the accumulation of double-stranded RNA (dsRNA), inhibiting ZBP1-mediated inflammasome activation and pyroptosis. Inhibition of the RNase activity of IRE1α using MKC8866 restores pyroptosis, enhances CD8 + T cell infiltration, and this sensitizes TNBC tumors to ICI and taxane-based chemotherapy. Additionally, in lung cancer, IRE1α–XBP1s signaling upregulates PTGES (encoding PGES1), promoting prostaglandin E₂ (PGE₂) production and immunosuppression. In PCa, activation of the IRE1α–XBP1s axis suppresses IFN responses and promotes the accumulation of TAMs within the TME. Combination therapy with MKC8866 and ICI reprograms the TME and enhances anti-tumor immunity

Increasing evidence suggests that in response to the stresses that they experience, both cancer cells and immune cells in the TME undergo chronic UPR activation with important implications [79, 155]. Activation of the UPR in cancer cells not only promotes cancer cell growth and malignancy, but also hinders anti-tumor immune responses in the TME (Fig. 3). The stress factors in the TME disrupt the ER folding capacity of infiltrating immune cells and lead to UPR activation that can help cancer cells to evade host immunity due to impaired anti-tumor immune responses [73].

Emerging evidence reveals that UPR and related cellular stress pathways play critical roles in regulating immune checkpoint molecules, such as programmed cell death protein 1 ligand 1 (PD-L1), thereby influencing tumor immune evasion and therapeutic outcomes [155, 156]. For example, BiP directly interacts with and stabilizes PD-L1 in TNBC cells, thereby enhancing immune evasion. This stabilization occurs in the ER, where BiP binding prevents PD-L1 degradation, leading to increased PD-L1 protein levels under ER stress conditions, including chemotherapy-induced stress [156]. Clinically, coordinate high expression of BiP and PD-L1 correlates with decreased relapse-free survival, underscoring the clinical relevance of this pathway [156]. In addition, IRE1α up-regulates PD-L1 expression via the NF-κB pathway upon ER stress in human melanoma cell lines, linking UPR signaling directly to immune checkpoint regulation [157]. Moreover, a recently published study has highlighted the role of the ISR pathway, a key arm of cellular stress responses closely related to the UPR, in coordinating the translational control of multiple immune checkpoints in lung cancer [158]. ISR activation robustly induces PD-L1 and CD155 expression through a mechanism involving bypass of inhibitory upstream ORFs in their 5’ UTRs, leading to enhanced translation of both immune checkpoint proteins [158]. Importantly, ISR activation correlated with tumor progression and suppression of T cell function, effects that could be reversed by combined blockade of PD-1, TIGIT, and ISR inhibition in the CMT167 mouse lung cancer model [158].

Recent research suggests that cancer cells experiencing ER stress responses can significantly affect the progression of malignancies by influencing the behavior of immune cells within the TME [73, 79, 159]. Early studies demonstrated that the activation of ER stress and UPR can hinder the expression of major histocompatibility complex class I (MHC-I) molecules on the cell surface, possibly facilitated by the overexpression of XBP1s and ATF6 [160]. Inducing ER stress in mouse EL4 lymphoma cells through exposure to palmitate or glucose deprivation triggered eIF2α-mediated suppression of protein synthesis [161]. This impaired the optimal loading of peptides onto MHC-I proteins, leading to compromised stability and abnormal localization at the cell surface [161]. Additionally, in response to ER stress, epithelial cells exhibited XBP1-dependent induction of microRNA miR-346, which downregulates the expression of transporter associated with antigen processing 1 (TAP1) in the ER, further impacting optimal peptide influx and MHC-I antigen loading [162].

The activation of specific ER stress sensors in cancer cells may also influence natural killer (NK) cell recognition of tumors. For example, the IRE1α-XBP1s pathway downregulated the expression of the NK group 2D (NKG2D) ligand MHC class I polypeptide-related sequence A (MICA) in human melanoma cells under ER stress conditions [163]. The level of MICA expression in melanoma samples correlated with XBP1s expression [163]. On the other hand, activation of the PERK-eIF2α pathway through pharmacological ER stress induced B7H6 expression (a ligand for the NK cell receptor NKp30) and rendered melanoma cells more susceptible to killing by chimeric antigen receptor (CAR) T cells that target B7H6 [164].

Moreover, recent findings indicate that ER stress in cancer cells can significantly alter the recruitment and function of immune cells within the TME [73]. Overactivation of IRE1α in TNBC cells led to increased production of pro-inflammatory and immunomodulatory cytokines, such as granulocyte–macrophage colony-stimulating factor (GM-CSF), CXC-chemokine ligand 1 (CXCL1), and interleukin-6/8 (IL-6/IL-8) [96]. Furthermore, disruption of IRE1α function promoted the remodeling of the TME in TNBC by enhancing pericyte levels, vascular normalization, and reducing the accumulation of cancer-associated fibroblasts (CAFs) and myeloid-derived suppressor cells (MDSCs) [165]. In glioblastoma cells, IRE1α-XBP1s signaling promoted the expression of IL-6, CC-chemokine ligand 2 (CCL2), and CXCL2, which attract macrophages and monocytes to the TME, which are often immunosuppressive [166]. Similarly, ER-stressed cancer cells may release factors that recruit and alter the function of myeloid cells, such as MDSCs, in the TME [73]. ER stress induction by thapsigargin in mice carrying CT26-derived colon tumors enhanced the recruitment and immunosuppressive activity of MDSCs, which was attenuated by compounds that relieve protein-folding stress [167].

ER stress in cancer cells can also affect the function of other immune cells, such as macrophages. Hepatocellular carcinoma (HCC) cells under ER stress release exosomes containing miR-23a-3p, which upregulate PD-L1 expression in macrophages, resulting in suppressive activity towards CD8 + T cells. ER stress response markers such as ATF6, PERK and IRE1α in human HCC specimens were associated with increased infiltration of CD68 + PD-L1 + macrophages and poor patient prognosis [168]. Moreover, when mouse myeloid cells were exposed to substances released by PCa cells experiencing ER stress, they triggered UPR activation, resulting in the initiation of a pro-tumorigenic and immunosuppressive phenotype. This phenomenon, known as "transmissible ER stress" (TERS), demonstrated the ability to upregulate immunosuppressive molecules such as Arginase 1 (Arg1) and prostaglandin E2 (PGE2) in DCs, resulting in impaired antigen presentation to cytotoxic T cells [169, 170]. Consequently, when DCs co-cultured in vitro with supernatants from ER-stressed cancer cells, they acquired an immunosuppressive phenotype that stimulated tumor growth in B16-F10 melanoma model after adaptive transfer into mice [170]. However, "carry-over effects" in TERS studies highlight the potential for residual, external ER stress inducers used in these experiments, such as thapsigargin, that can inadvertently activate ER stress in recipient cells, rather than through authentic paracrine signaling. This complicates the interpretation of TERS, since distinguishing between genuine ER stress transmission and direct chemical induction is challenging, especially when identifying true mediators of cell-to-cell stress signaling [171].

An additional key impact of ER stress in cancer cells on the TME is that it can modulate T cell mediated anti-tumor immunity and impact tumor growth, metastasis, and response to immunotherapy (Table 1 and 2) [73]. In a recent study, immunocompromised mice that received orthotopic injection of Coactivator-Associated Arginine Methyltransferase 1 (CARM1)-high ovarian cancer cells showed enhanced therapeutic responses to the IRE1α RNase inhibitor B-I09 compared to mice with CARM1-low ovarian tumors [192]. Furthermore, an additive effect was observed when combining B-I09 with programmed cell death 1 (PDCD1 or PD-1) blockade in tumor-bearing immunocompetent mice [192]. In another study, in mouse models of pancreatic cancer, forced overexpression of XBP1s in cancer cells, coupled with systemic T cell depletion, promoted the growth of macrometastatic lesions [200]. In contrast, knocking down XBP1 in melanoma cells enhanced the effectiveness of anti-PD-1 immune checkpoint inhibition (ICI). Furthermore, reduced expression of UPR target genes such as XBP1s, ATF4, and BiP was observed in the B2905 mouse melanoma model upon anti-cytotoxic T lymphocyte-associated antigen 4 (CTLA-4) ICI [193]. Importantly, lower levels of XBP1s, ATF4, and BiP expression in pre-treatment tumor biopsy samples significantly correlated with better treatment responses and prolonged survival in different groups of melanoma patients undergoing anti-CTLA-4 therapy [193]. Similar findings were observed in studies involving colorectal cancer where the expression of ATF6 was associated with reduced disease-free survival [201]. Taken together, these studies highlight the ability of ER-stressed cancer cells to orchestrate various mechanisms within the TME to evade the immune response and facilitate malignant progression.

**Table 1:** ER stress-induced modulation of immune responses in the TME

Immune cell type	Pathway/protein	Effect/function	Specific observations/mechanism	Model	Reference
Macrophages	IRE1α-XBP1s	Increased immunosuppressive functions, reduced phagocytic ability, M2-like polarization	Increased VEGFA, IL4, IL6, SIRPA, THBS1, ARG1, PD-L1	Colorectal cancer and melanoma	[172–174]
Macrophages	PERK-ATF4	Increased immunosuppressive functions and M2-polarization	Increased PSAT1, GLUT1, IL-10	Melanoma and GBM	[175, 176]
Dendritic cells	IRE1α-XBP1s or IRE1α	Increased lipid biosynthesis and uncontrolled buildup of lipid droplets in the cytosol, and downregulation of MHC-I molecules through RIDD	Reduced ability of DCs to present antigens to T cells	Ovarian, colorectal, and breast cancer	[114, 177]
Neutrophils	IRE1α or ER stress	Promote immunosuppression, T cell exhaustion, and tumor progression	Elevated ER stress markers (BiP, CHOP, ATF6), suppression of T cells via CCL5 and Nectin2	Autochthonous HGSOC or PDAC (mouse and human models)	[178, 179]
Myeloid-derived suppressor cells or polymorphonuclear MDSCs	PERK-ATF4, NRF2 or IRE1α and ATF6	Increased mitochondrial and immunosuppressive activities	Reduced infiltration of CD8 + T cells and NK cells	Lewis lung carcinoma, melanoma, breast, and colorectal cancer	[180–182]
Splenic hematopoietic stem and progenitor cells	PERK-ATF4, C/EBPβ	Increased differentiation of HSPCs to differentiation into MDSCs	Reduced infiltration of CD8 + T cells	Hepatocellular carcinoma	[183]
T cells	IRE1α-XBP1s	Reduced T cell effector functions and anti-tumor immunity	Reduced glutamine uptake and mitochondrial respiration, increased expression of PD-1, TIM-3, 2B4 and LAG3	Ovarian cancer, melanoma, and colorectal cancer	[184, 185]
T cells	IRE1α-XBP1s	Reduced metabolic fitness and T cell effector function	Reduced lipid uptake, mitochondrial respiration and cytotoxicity	Ovarian cancer	[186]
T cells	PERK-ATF4, CHOP, T-bet	Reduced mitochondrial and T cell effector function	Inhibition of IFN-γ production through TBX21 and increased mitochondrial ROS	Melanoma and sarcoma	[187, 188]
Natural killer cells	IRE1α–XBP1s	NK cell proliferation and survival	Increased NK cell infiltration, effector functions, anti-tumor immunity	Melanoma and PCa	[82, 189, 190]
B cells	IRE1α–XBP1s	Enhanced immunosuppressive activities of MDSCs	Increased secretion of IgM and accumulation of MDSCs	Chronic lymphocytic leukemia and Lewis lung carcinoma	[191]

**Table 2:** Targeting UPR to enhance immune check-point inhibition therapies

Combination	UPR target and agent	Model	Findings	Reference
B-I09 + anti-PD-1	Small molecule IRE1α inhibitor	Orthotopic UPK10 syngeneic mouse ovarian cancer	Increased infiltration of CD4 + and CD8 + T cells, and B cells, with decreased MDSCs and tumor growth	[192]
sh-XBP1 + anti-PD-1	shRNA- mediated XBP1 knockdown in YUMM1.7 cells	YUMM1.7	Reduced tumor growth	[193]
α-T-K + anti-PD-1	Encapsulated IRE1α inhibitor KIRA6 into a nanoemulsion containing α-tocopherol	LLC syngeneic mouse Lewis lung cancer	repolarize M2-TAMs under hypoxia	[194]
GSK2656157 + anti-PD-1	Small molecule PERK inhibitor	B16-F10 syngeneic mouse melanoma and MOC2 syngeneic mouse oral squamous cell carcinoma	Increased tumor infiltrating lymphocytes and reduced TAMs Increased infiltration of CD8 + T cells, reduced TAMs and lymph node metastasis	[175, 195]
*Eif2ak3*^*fl/fl*^*cre*^+^ mice + α4-1BB	In the host mice site-specific deletion of PERK in myeloid cells	Orthotopic SB28 syngeneic mouse GBM	Increased infiltration of effector T cells and cancer survival	[176]
G9668 + anti-PD-L1	Small molecule IRE1α inhibitor	4T1 syngeneic mouse breast cancer	Increased infiltration of DCs and CD8 + T cells, and their activation	[177]
Ern1^f/f^cre^+^Mrp8 mice bearing autochthonous HGSOC + anti-PD-1	In the host mice site-specific deletion of IRE1α in neutrophils	Autochthonous HGSOC generated by introducing plasmids that overexpress *myc* and target p53 into the ovary using in vivo electroporation	Elevated PD-1 levels on tumor infiltrating T cells and increased PD-L1 expression on myeloid cells, increased overall survival	[178]
AMG-44 + anti-PD-L1	Small molecule PERK inhibitor	B16-F10 syngeneic mouse melanoma	Reduced the immunosuppressive activity of MDSCs and increased infiltration of effector CD8 + T cells	[180]
KIRA8 + anti-PD-1 or sh-XBP1 knockdown cancer cells + anti-PD-1	Small molecule IRE1α inhibitor or shRNA mediated XBP1 knockdown in B16-OVA cells	B16-OVA and MC-38-OVA syngeneic mouse melanoma and colon	Increased infiltration of effector CD8 + T cells and reduced MDSCs	[196]
MKC8866 + anti-PD-1	Small molecule IRE1α inhibitor	Myc-CaP, Myc-CaP PTEN KO, and RM-1 syngeneic mouse PCa models	Increased infiltration of CD8 + T cells and NK cells, reduced abundance of TAMs with M1-like phenotype with increased interferon responses	[82]
MKC8866 + docetaxel + anti-PD-1	Small molecule IRE1α inhibitor	2250L, 2153L, 2331L Trp53 − / − genetically engineered TNBC mouse model lines	Conversion of PD-L1 negative tumors to PD-L1 high immunogenic tumors, increased infiltration of effector CD8 + T cells and proliferation, reduction in PMN-MDSCs, increased C1 MHC-II high macrophages and decreased C0 Trem2 + macrophages	[197]
GSK2606414 + anti-PD-1	Small molecule PERK inhibitor	MCA-205-OVA syngeneic mouse sarcoma	Reduced tumor growth and increased survival	[188]
PERK KO cancer cells + anti-PD-1	CRISPR-Cas9 mediated knock-out of PERK gene in B16-F10 cells	B16-F10 syngeneic mouse melanoma	Decreased tumor growth	[198]
ERO1α KO cancer cells + anti-PD-1 or KIRA6 + anti-PD-1	CRISPR-Cas9 mediated knock-out of ERO1α in LLC, B16-F10, and MC-38 cells or Small molecule IRE1α inhibitor	LLC, B16-F10, and MC-38 syngeneic mouse Lewis lung carcinoma, melanoma, and colon or MC-38 syngeneic mouse colon	Augmented anti-tumor effects	[199]

Although activation of UPR pathways are generally considered to promote immunosuppression in the TME, it is important to note that UPR can also be activated in immune hot tumors such as in melanoma and non-small cell lung cancer (NSCLC) [202]. On the other hand, recent studies in syngeneic mouse models of these cancers indicated that loss of PERK or IRE1α indeed activate anti-tumor immunity in the TME [198, 203], suggesting that UPR activation in the immune hot tumors could also promote immunosuppression. Nevertheless, exploring how UPR activation influences cancer-immune cell interactions, tumor antigen presentation, and immune checkpoint regulation in immune hot tumors could redefine our understanding of UPR's role in cancer immunity. For example, do UPR-driven changes in antigen presentation or processing influence the efficacy of ICI in immune hot tumors? Investigating this aspect could shed light on novel mechanisms that link UPR activation and immune modulation, potentially with translational impact to enhance anti-tumor immune responses in patients who are not responsive to ICI therapy.

## The role of UPR in immune cell function and immune suppression

The disturbance of normal ER homeostasis in immune cells that infiltrate tumors has been identified as a significant factor in facilitating tumor promotion and the ability of the cancer cells to evade the immune system. The hostile environment within the TME plays a critical role in maintaining prolonged ER stress in the infiltrating immune cells. The altered nutrient composition and its limited availability, high metabolic demand, accumulation of ROS and acidic conditions in the TME all affect protein folding capacity and trigger persistent ER stress in tumor-infiltrating leukocytes (TILs) [204]. Recent studies have shown that TILs exhibit persistent activation of ER stress signaling pathways, leading to modulation of transcriptional and metabolic programs specific to immune cells [73, 97, 205, 206]. Below, we provide a summary of these recent findings and highlight the impact of ER stress on various immune cells within the TME (Table 1). We also summarize recent data suggesting that targeting UPR signaling can increase the efficacy of ICI (Table 2).

### Tumor-associated macrophages

Tumor-associated macrophages (TAMs) represent a prominent myeloid population in the TME of various cancer types. They have been implicated in promoting angiogenesis, therapy resistance, and immunosuppression [207, 208]**.** Recent studies have shown that ER stress and UPR activation programs macrophages into immunosuppressive phenotypes. In vitro studies have indicated that macrophage-intrinsic IRE1α was associated with the promotion of cancer cell invasion through the secretion of cathepsin [209]. Other investigations have revealed that TAMs obtained from patients with colorectal cancer, or from a mouse model of colitis-associated cancer, exhibited increased XBP1s levels compared to human peripheral blood monocytes and macrophages from mouse spleen, respectively [172]; these data indicate that IRE1α signaling is activated in TAMs.

In bone marrow-derived macrophages (BMDMs) exposed to conditioned media from colorectal cancer cells, the presence of XBP1s directly stimulated the expression of VEGFA, IL4, IL6, and ligands associated with “don't eat me” signals, such as signal regulatory protein α (SIRPα) and thrombospondin 1 (THBS1) [172]. Consistently, absence of XBP1s led to an enhancement in macrophage phagocytic capacity and hindered tumor development in a mouse model of colorectal cancer; however, the specific molecular mechanisms involved remain unclear [172]. Furthermore, in the B16-F10 melanoma model, in response to conditioned media from ER stressed cells, BMDMs showed increased expression of pro-inflammatory cytokines and immunosuppressive factors such Arg1 and PD-L1; inhibition of IRE1α signaling reversed these effects in BMDMs [173]. Interestingly, B16-F10 tumor-bearing mice with selective deficiency of IRE1α in macrophages showed improved survival, whereas the effects observed were not replicated when XBP1s was ablated specifically in macrophages [173]. Consistently, macrophages required active IRE1α-XBP1s signaling to facilitate the optimal production of pro-inflammatory cytokine IL-6 in response to stimulation through Toll-like receptors (TLRs) [210]. In addition, human macrophages required IRE1α for the expression of IL-6, IL-10, PD-L1, and VEGF when infected with Kaposi's sarcoma herpesvirus [211].

The potential translation of this knowledge into the clinic is exemplified by a study wherein the IRE1α small molecule inhibitor KIRA6 was encapsulated in a nanoemulsion containing α-tocopherol (α-T-K) that effectively inhibited both ER stress and oxidative stress [194]. α-T-K reprogrammed M2 macrophages under hypoxia by increasing glycolysis and reducing fatty acid oxidation, leading to delayed tumor growth in the 4T1 BCa model and enhanced the efficacy of PD-1 ICI in the LLC lung cancer model. Thus, coordinately targeting UPR and oxidative stress may be a potential strategy for anticancer therapy.

Furthermore, a recent study demonstrated that when co-cultured with melanoma cells, both TAMs and BMDMs showed increased activation of UPR and a shift toward an immunosuppressive polarization phenotype, again demonstrating the inhibitory effects of the UPR in the TME immune landscape [174]. Mechanistically, cancer cells utilized β-glucosylceramide to activate the Ca^2+^-dependent lectin receptor (Mincle) on macrophages, leading to modifications in ER membrane lipid composition. This alteration triggered the IRE1α–XBP1s signaling pathway. Notably, when tumor-bearing mice were treated with a liver X receptor (LXR) agonist, expression of lysophosphatidylcholine acyltransferase 3 (LPCAT3) was induced, which counteracts lipid-induced ER stress by synthesizing phosphatidylcholine enriched with unsaturated fatty acids; this in turn decreased XBP1s levels in TAMs, resulting in improved survival of the tumor-bearing mice [174].

Recent research has also shown that TAMs polarize towards the tumor promoting M2-like phenotype, characterized by elevated PERK signaling compared to naïve or anti-tumorigenic M1-like macrophages [175]. In the B16-F10 melanoma model, PERK-deficient macrophages exhibited impaired M2 polarization [175] coincident with lower levels of ATF4-mediated stimulation of serine biosynthesis, which supported M2 polarization. This was due to lower levels of phosphoserine aminotransferase 1 (PSAT1) expression in PERK-deficient TAMs, an enzyme crucial for serine biosynthesis, leading to reduced mitochondrial fitness and significant epigenetic alterations [175]. Genetic or pharmacological inhibition of PERK in TAMs resulted in delayed tumor growth, increased infiltration of T cells, and enhanced efficacy of anti-PD-1 treatment [175]. Consistently, in a recent study, PERK + macrophages were found to drive resistance to neoadjuvant anti-PD-1 immunotherapy in oral squamous cell carcinoma (OSCC) patients, particularly within lymph node (LN) metastases [195]. These macrophages, enriched in LN lesions, created an extracellular matrix-dense microenvironment through interactions with fibroblasts, which hindered T cell-mediated cytotoxicity [195]. Notably, in a preclinical orthotopic OSCC mouse model, small molecule PERK inhibitor GSK2656157 combined with anti-PD-1 therapy reprogrammed the TME and disrupted immunosuppressive macrophage–fibroblast interactions and enhanced CD8+ T cell infiltration, leading to regression of both primary tumors and metastatic LNs [195].

Immunosuppressive macrophages also hindered anti-tumor immunity in glioblastoma (GBM). A recent study showed that monocyte-derived macrophages (MDMs) outnumber microglia in late-stage GBM tumors and suppress T cell activity through high glycolysis and lactate production, driven by GBM-derived factors [176]. This process is regulated by the PERK-ATF4 axis, which promotes GLUT1 expression and histone lactylation, leading to IL-10 production and immunosuppression. Inhibition of glycolysis or PERK deletion in MDMs reduced these immunosuppressive effects, enhanced T cell infiltration, delayed tumor growth, and improved the effectiveness of 4-1BB (also known as CD137 or TNF receptor superfamily member 9 (TNFRSF9)) based immunotherapy [176].

### Dendritic cells

ER stress response is known to dampen the function of intratumoral DCs [97, 212]. For example, in ovarian cancer, activation of ER stress and the IRE1α-XBP1s pathway occurs in intratumoral DCs, driven by the accumulation of ROS and generation of byproducts from lipid peroxidation [114]. Consequently, XBP1s induced a transcriptional program promoting lipid synthesis, resulting in uncontrolled buildup of lipid droplets in the cytosol that impaired antigen presentation to T cells [114]. In mouse models of ovarian cancer, genetic elimination of IRE1α or XBP1s enhanced the function of intratumoral DCs, leading to improved adaptive immune responses and prolonged survival of tumor bearing mice [114]. Subsequent studies demonstrated that treating mice with α-T-K, inhibiting both ER stress and oxidative stress as introduced above, in the ID8 mouse ovarian cancer model resulted in reduced tumor growth [213].

Recent research found that antigenic peptides directly activate IRE1α in DCs, resulting in the downregulation of MHC-I molecules through RIDD [177]. By inhibiting IRE1α function in DCs, the effects of RIDD were alleviated, and this led to an increase in the ability of DCs to present antigens to T cells [177]. In the same study, systemic pharmacological inhibition of the IRE1α kinase activity using the small molecule inhibitor G9668 significantly improved anti-tumor responses and enhanced the effectiveness of anti-PD-L1 ICI in mouse BCa models [177]. However, the specific mechanisms underlying these therapeutic effects, such as the restoration of intratumoral DC function and/or the enhancement of adaptive immunity, are yet to be determined and require further investigation.

Another line of evidence indicating the role of UPR in DC function is provided by DCs lacking BAT3, an adaptor protein that binds to TIM-3 on T cells. DCs without BAT3 displayed excessive activation of UPR which led to a tolerogenic phenotype that hampered the T cell immune responses against tumors in mice [214]. The activation of the UPR in BAT3-deficient DCs was attributed to the role of BAT3 as an ER chaperone involved in the quality control of newly synthesized proteins. DCs isolated from spleen and lymph nodes of MC38 tumor-bearing mice displayed low levels of BAT3 expression compared to splenic naïve DCs, which were proposed to promote tumor growth [214]. Notably, bone marrow-derived DCs that lack BAT3 showed reduced expression of co-stimulatory molecules, which could be restored by treatment with the ER stress-alleviating chemical chaperone 4-Phenylbutyric acid (4BPA) or the IRE1α inhibitor 4μ8C [214]. Furthermore, UPR activation caused by BAT3 deficiency altered the metabolism of DCs, which led to redirection of acetyl-CoA towards the production of immunosuppressive glucocorticoids [214]. These data collectively highlight the possibility of inhibiting IRE1α signaling in intratumoral DCs as a way to enhance anti-tumor immune responses and improve the efficacy of cancer immunotherapy. Further research is required to assess this translational potential and also delineate the possible roles of PERK and ATF6 signaling on DC function and regulation of anti-tumor immune responses.

### Neutrophils

In many cancer patients, tumour-associated neutrophils (TANs) typically adopt an immunosuppressive, pro-tumorigenic role [215–217]. A recent study using a mouse model of high-grade serous ovarian carcinoma (HGSOC) demonstrated that tumors reprogram neutrophils to suppress T cell–mediated anti-tumor immunity by activating IRE1α [178]. Compared to neutrophils in non-tumor tissues, TANs showed increased expression of ER stress markers [178]. Deleting IRE1α specifically in neutrophils slowed tumor growth and improved survival by enabling early T cell responses. Additionally, IRE1α loss sensitized tumors to anti-PD-1 therapy, leading to tumor regression and long-term survival in about half of treated mice [178]. In pancreatic ductal adenocarcinoma (PDAC), both human and mouse TANs displayed elevated ER stress marker genes such as BiP, CHOP, and ATF6 [179]. These TANs promoted cancer cell migration and T cell exhaustion via CCL5 and Nectin2 [179]. Alleviating ER stress with 4BPA reduced these effects and limited tumor progression [179]. Overall, targeting ER stress in neutrophils may enhance both natural and immunotherapy-driven anti-cancer responses with potential translational applications.

### Myeloid-derived suppressor cells

MDSCs are composed of immature myeloid cells that have T cell suppressive activities. PERK plays a key role in maintaining the integrity of mitochondrial DNA (mtDNA) within MDSCs and their immunosuppressive functions in various tumor models [180]. Targeting PERK disrupted mitochondrial functionality in a NRF2-dependent manner, resulting in the release of mtDNA into cytosol, which led to reprogramming of MDSCs through the activation of stimulator of interferon signaling (STING) [180]. Consistently, the small molecule PERK inhibitor AMG-44 reduced the immunosuppressive activity of MDSCs, promoted the expansion of tumor infiltrating CD8 + T cells expressing interferon gamma (IFNγ) and synergized with anti-PD-L1 ICI [180]. In another study, treatment of 4T1 BCa tumors that have high chromosomal instability with AMG-44 led to a significant reduction in granulocytic MDSCs and a corresponding increase in the infiltration of NK cells and CD8 + T cells [181].

The immunosuppressive function of MDSCs is also diminished by targeting downstream factors of PERK, such as CHOP or ATF4, in different mouse tumor models [218]. Ablation of CHOP or ATF4 in the tumor stroma resulted in reduction of TME-induced CCAAT/enhancer-binding protein-β (C/EBP-β) signaling which led to decreased phospho-STAT3 and IL-6 levels, thereby attenuating the immunosuppressive properties of MDSCs [218]. Another study found that the immunosuppressive function of polymorphonuclear MDSCs (PMN-MDSCs), but not monocytic MDSCs, is dependent on IRE1α and ATF6 [182]. Interestingly, the absence of either of these ER stress sensors in myeloid cells (enabled by the use of conditional KO mouse models) led to a delay in tumor growth in the MC38 mouse model of colon cancer; however, it did not affect the tumor progression of Lewis lung carcinoma (LLC) [182]. Notably, XBP1s activation in cancer cells promoted the production and release of cholesterol, which in turn activated MDSCs and contributed to tumor suppression. Genetic targeting of XBP1 or pharmacological inhibition of IRE1α kinase activity using KIRA8 led to reduced cholesterol levels and MDSC abundance, resulting in activation of effective anti-tumor immunity in mouse cancer models [196]. Activation of PERK in splenic hematopoietic stem and progenitor cells (HSPCs) promoted tumor progression in a hepatoma model [183]. The underlying mechanism involves IL-6 produced by the stroma, which induces PERK-ATF4-C/EBPβ signaling in HSPCs, leading to their differentiation into MDSCs [183]. Targeted delivery of PERK inhibitors, such as AMG-44 or GSK2606414, to the spleen using a micro-osmotic pump system resulted in effective tumor control, significantly more than direct delivery to the tumors, emphasizing the detrimental role of PERK activation in splenic MDSCs [183].

### T cells

The presence of stress factors within the TME plays a critical role in inhibiting T cell function that contributes to tumor growth [97, 219, 220]. Aberrant activation of the IRE1α-XBP1s pathway in intratumoral T cells inhibited antitumor activity by modulating their mitochondrial function and fitness, which in turn limited protective T-cell responses in a metastatic ovarian cancer model [184]. Selective deletion of XBP1 or IRE1α in mouse CD4^+^ T cells improved their effector function and increased survival. Consistently, recent findings in mouse ovarian cancer models documented that ER stress repressed expression of cytoskeletal organizer transgelin 2 (TAGLN2) in CD8+ T cells, which impaired fatty acid uptake, mitochondrial respiration, and cytotoxic function [186]. TAGLN2 normally supports the function of fatty-acid-binding protein 5 (FABP5), which is essential for importing and trafficking lipids to fuel mitochondrial metabolism in activated T cells. Restoring TAGLN2 expression in ER stressed CD8 + T cells not only boosted their metabolic resilience but also enabled enhanced antitumor capacity, underscoring the therapeutic potential of targeting ER stress responses to optimize T-cell function in solid tumors by activating the TAGLN2–FABP5 axis [186].

Another study using the B16-F10 melanoma model showed that cholesterol accumulation in intratumoral CD8+ T cells led to expression of inhibitory receptors on their surface, such as PD-1, TIM-3, and LAG3, which contributed to T cell exhaustion [185]. Furthermore, genetic inhibition of XBP1 or pharmacological targeting of IRE1α using STF-083010 enhanced CD8+ T cell anti-tumor activity that resulted in reduced lung metastasis of tumor bearing mice [185]. In mouse syngeneic melanoma and colon cancer models, the combination treatment with the IRE1α inhibitor KIRA8 and anti-PD-1 significantly enhanced anti-tumor immune responses [196]. Comparable outcomes were observed when XBP1 was silenced in B16-OVA cells (a melanoma line expressing ovalbumin), followed by anti-PD-1 ICI [196]. A recent study in a NSCLC mouse model found that upon IRE1α targeting, the number of CD8 + T cells were increased within the TME, resulting in enhanced adaptive immunity [203].

Consistent with these results, we recently found that genetic deletion or small molecule inhibition of the IRE1α-XBP1s signaling led to an increased response to anti-PD-1 ICI therapy in various syngeneic PCa mouse models [82]. CRISPR/Cas9-mediated genetic targeting of IRE1α or treatment with the small molecule IRE1α RNase inhibitor MKC8866 (ORIN10010), which is currently in clinical trials, significantly reprogrammed the TME; this resulted in reversal of immunosuppression, enhanced NK and CD8 + T cell infiltration, boosted interferon responses, and improved effectiveness of anti-PD-1 therapy in multiple PCa mouse models. Additionally, we identified a novel TAM gene signature linked to poor PCa survival, which was significantly reduced in mouse PCa models treated with MKC8866+anti-PD1 therapy [82].

Complementing these findings in PCa, a recent study in TNBC has uncovered that IRE1α plays a key role in limiting the immune-stimulating effects of chemotherapy in immunologically 'cold' tumors. RNase activity of IRE1α prevented the accumulation of taxane-induced double-stranded RNA (dsRNA) through RIDD, which blocked pyroptosis by inhibiting the NLRP3 inflammasome [197]. Inhibition of IRE1α in TNBC allowed taxane chemotherapy to increase dsRNA levels that, when sensed by Z-DNA binding protein 1 (ZBP1), activated the NLR family pyrin domain containing 3 (NLRP3)-Gasdermin D (GSDMD) pathway, converting PD-L1-negative, ICI-resistant TNBC tumors into PD-L1-high, immunogenic ones that are highly responsive to ICI [197]. Together, these studies suggest that IRE1α inhibition could enhance the effectiveness of immunotherapies across different cancer types, by addressing distinct immunosuppressive mechanisms. Further research is needed to explore the translational potential of targeting IRE1α in this context for additional cancer types.

PERK signaling also significantly affects T cell function in the TME. The activation of PERK downstream target gene CHOP in intratumoral T cells has been found to hinder effector function of T cells in mouse ovarian and other cancer models [187]. Moreover, elevated expression of CHOP in infiltrating T cells was correlated with unfavorable clinical outcomes in ovarian cancer patients [187]. Deletion of CHOP in CD8+ T cells resulted in the restoration of anti-tumor immunity by relieving the repression of T-bet (TBX21), a key regulator of type I anti-tumor immune responses [187]. Persistent activation of PERK in tumor antigen-specific T cells was associated with compromised mitochondrial function; consistently, inhibiting PERK activity in T cells led to a reduction in mitochondrial ROS, resulting in enhanced anti-tumor immunity [188]. Furthermore, PERK inhibition with the small molecule inhibitor GSK2606414 reduced the mitochondrial ROS levels in CD8+ tumor infiltrating T cells and enhanced the efficacy of anti PD-1 immunotherapy response in a mouse sarcoma model [188]. In another study on melanoma, PERK loss in cancer cells promoted immunogenic cell death and IFN production in DCs, converting them into inflammatory cells that then stimulate the infiltration of CD8 + T cells, resulting in enhanced anti-tumor immunity. In addition, in a syngeneic mouse model of melanoma PERK deletion sensitized the tumors to anti-PD1 ICI and resulted in stronger anti-tumor immune responses [198].

Elevated expression of Endoplasmic Reticulum Oxidoreductase 1 Alpha (ERO1A) in lung cancer patients was linked to an increased risk of recurrence after neoadjuvant immunotherapy [199]. ERO1A mediated an immune-suppressive TME, reducing the effectiveness of PD-1 blockade. Ablating ERO1A in various syngeneic mouse models of cancer (colorectal, lung, and melanoma) impaired activation of the IRE1α-XBP1s pathway, induced immunogenic cell death, and enhanced the efficacy of anti-PD-1 therapy. Additionally, the small molecule IRE1α inhibitor KIRA6 further enhanced anti-PD-1 therapy and boosted anti-tumor immunity in the MC38 model that expresses wild-type ERO1A [199].

The influence of UPR activation on the functionality of regulatory T cells (Tregs) in cancer remains uncertain, but there are indications that ER stress could affect the biology of this specific subset of T cells as well. Studies suggest that induction of ER stress in both human and mouse Tregs led to increased production of TGF-β and IL-10; however, this effect was diminished when PERK or eIF2α was inhibited [221, 222]. Genetic targeting of ATF4 enhanced FOXP3 mRNA expression in mouse CD4 + T cells cultured in an oxidizing environment under conditions that promote Treg differentiation [223]. In addition, genetic deletion of IRE1α decreased Treg levels in the PCa TME in multiple syngeneic models [82]. Similarly, Treg infiltration was significantly decreased in a NSCLC mouse model [203]. These findings underscore the functional relevance of ER stress signaling for Treg function that should be studied in greater detail in the future.

### Natural killer cells

The activation of IRE1α–XBP1s signaling pathway has a significant role in facilitating the optimal proliferation of NK cells, partly by promoting MYC expression [189]. Consequently, in various models of viral infection and cancer, the absence of IRE1α in NK cells led to reduced infiltration of NK cells, increased tumor growth, and decreased overall survival [189]. A recent study showed that XBP1s is crucial for the survival of human NK cells mediated by interleukin-15 (IL-15) and AKT signaling [190]. Mechanistically, XBP1s targeted the anti-apoptotic gene PIM-2 to regulate NK cell survival; in addition, XBP1s enhanced NK cell effector functions and antitumor immunity by recruiting T-bet to the IFN-γ promoter region [190]. In contrast, the number of NK cells in the TME was increased upon systemic IRE1α inhibition with MKC8866 in syngeneic mouse models of PCa [82], suggesting that IRE1α signaling may have context dependent differences on NK cell survival and activity. Additional studies are necessary to address whether IRE1α inhibitors can negatively affect NK cell function and survival in different cancer models.

### B cells

XBP1s is essential for the development of plasma cells and secretion of immunoglobulin M (IgM) [224]. Genetic deletion of XBP1 led to phosphorylation of IRE1α at S729, resulting in activated RIDD activity that hindered the expression of the secretory heavy chain μ; as a consequence, IgM secretion by B cells was compromised [225, 226]. Recent findings have revealed that secretory IgM enhances the immunosuppressive activities of MDSCs in chronic lymphocytic leukemia (CLL) and LLC syngeneic mouse lung cancer models; in addition, the negative impact of secretory IgM was counteracted by the genetic loss of XBP1s, leading to effective T cell-mediated control of the tumor [191]. Further research is required to address how ER stress in the TME can modulate the function and abundance of infiltrating B cells in the TME.

Taken together, there is strong evidence that UPR signaling critically alters immune cell function in the TME. This opens up possibilities for novel therapeutic interventions, but also brings up questions as to whether systemic inhibition of UPR signaling may have opposing effects in cancer cells versus immune cells that may affect efficacy of treatment and their translational value. Further research is required to assess this possibility.

## Conclusions

During tumor formation, cancer cells hijack the normal stress response pathways to adapt to various stressors that impact the TME. Solid tumors rely on UPR signaling to survive under intra- and extra-cellular stress conditions. A key general aspect of some cancer types, such as PCa and BCa, is that they have low mutational burden that limit the presence of tumor-associated antigens that normally stimulate antitumor immune responses [227]. This makes these tumors immunologically ‘cold’ and unresponsive to ICI. Furthermore, in these cases the TME exhibits active immune suppression, involving infiltration of immunosuppressive cell types such as M2-polarized macrophages, MDSCs, and Tregs. These cells produce immunosuppressive cytokines and express immune checkpoints, hindering effective antitumor immunity [228–233]. Hypoxia and a protumorigenic cytokine profile further contribute to immune checkpoint upregulation [234, 235].

To address these challenges, therapeutic strategies must consider the immunosuppressive TME and the low mutational burden of tumors to make them immune responsive. To achieve a therapeutic response, innovative approaches should target both the cancer cells in the tumor and all other cells in the TME, aiming to enhance cytotoxic T-cell infiltration, promote effector function, and reduce immune suppression.

Significant data from recent studies in multiple tumor types have shown that the UPR plays a key role in both promoting cancer cell growth as well as establishing an immunosuppressive TME. The small molecule inhibitors that target the UPR have recently become available and opens up the possibility for translational applications. For example, preclinical studies have demonstrated that the IRE1α RNase inhibitor MKC8866 has potent anti-tumor activity in various cancer types, including multiple myeloma, BCa, and PCa; in addition, it synergizes with anti-cancer drugs that are in the clinic, such as, enzalutamide, abiraterone, cabazitaxel, docetaxel, and paclitaxel [81, 83, 96]. Furthermore, MKC8866 increased ICI efficacy in various mouse models [82, 197]. Thus, it would be valuable to investigate whether coupling MKC8866 with targeted or chemotherapies and checkpoint inhibitors can enhance clinical management and reverse resistance, not only for PCa but also for other cancer types, such as TNBC and PDAC.

Small molecule inhibitors that target IRE1α and PERK have demonstrated promising immunomodulatory effects in various preclinical tumor models. Inhibitors of IRE1α, such as B-I09, G9668, KIRA8, and MKC8866 enhance the infiltration and activation of CD8 + T cells, DCs, and NK cells while repolarize TAMs and reduce immunosuppressive MDSC populations [82, 177, 192, 194, 196, 197]. Similarly, PERK inhibitors such as GSK2656157, GSK2606414, and AMG-44 mitigate immune suppression by disrupting mitochondrial integrity in MDSCs and reduce T cell dysfunction, which improves CD8 + T cell infiltration and anti-PD-1/PD-L1 responses [175, 180, 181, 188, 195]. Together, these observations support IRE1α and PERK inhibition as a promising strategy to reprogram the immunosuppressive TME and increase the responsiveness to immunotherapy.

One potential drawback of IRE1α and PERK inhibition is that their earlier versions had significant issues, including poor selectivity, pancreatic toxicity linked to type-I IFN expression, activation of GCN2-mediated integrated stress response pathway and inhibition of receptor interacting serine/threonine kinase 1 (RIPK1) and KIT receptor tyrosine kinase (KIT) [236–241]. Newer versions of these inhibitors have better selectivity and safety, such as MKC8866, HC-5404 and NMS-03597812, that are currently being tested in phase I clinical trials for advanced cancer (NCT03950570), solid tumors (NCT04834778) and relapsed or refractory multiple myeloma (NCT05027594), respectively. For example, in a recent study, combining HC-5404 PERK inhibitor with VEGF receptor tyrosine kinase inhibitors, such as Axitinib and Lenvatinib, significantly enhanced the anti-tumor and antiangiogenic effects of these drugs in multiple renal cell carcinoma xenograft tumor models [242]. Furthermore, HC-5404 and anti-PD-1 combination therapy significantly enhanced antitumor efficacy in a syngeneic mouse bladder cancer model, correlated with increased immune activation markers on tumor cells and TAMs, including IFNAR1 and calreticulin, and reduced immunosuppressive activity of MDSCs [243]. Since HC-5404 showed acceptable tolerability and safety profile in the clinical setting with advanced solid tumors [244], it could be interesting to test whether combining HC-5404 with anti-PD-1 therapy or VEGF receptor tyrosine kinase inhibitors might be effective in cancer patients who are unresponsive or resistant to these treatments. Since HC-5404 demonstrated acceptable tolerability and safety profile in clinical settings with advanced solid tumors, it would be interesting to test whether combining HC-5404 with anti-PD-1 or VEGF receptor tyrosine kinase inhibitors could benefit cancer patients, including those with PCa, which were unresponsive or resistant to these treatments.

While UPR inhibition holds promise for disrupting tumor adaptation to stress and reprogramming the immunosuppressive TME, it is important to consider potential compensatory mechanisms that may limit long-term efficacy. Chronic suppression of IRE1α or PERK signaling may trigger adaptive responses that restore cellular homeostasis through alternative pathways such as AMP-activated protein kinase (AMPK), autophagy, or ISR. Additionally, prolonged inhibition of UPR arms could potentially lead to apoptosis resistance in tumor cells or immune escape mechanisms. Therefore, future studies should evaluate the impact of sustained UPR inhibition on tumor plasticity and immune evasion, and explore rational combinations that prevent or counteract these adaptations to maintain therapeutic benefit.

Overall, the integration of UPR inhibitors such as MKC8866 and HC-5404 with existing therapies presents a promising avenue for overcoming the immune resistance and low mutational burden characteristic of immunologically cold tumors, potentially leading to more effective treatment strategies and improved patient outcomes. In the future, it is necessary to explore the potential of combination therapies involving UPR inhibitors with ICI, bispecific antibodies, immune-modulating agents, and targeted therapies to determine if they can be instrumental to effectively treating immunologically cold tumors and improving patient outcomes.

## Data Availability

No datasets were generated or analysed during the current study.

## Abbreviations

UPR: The unfolded protein response
TME: Tumor microenvironment
ER: Endoplasmic reticulum
IRE1α: Inositol-requiring protein 1α
PERK: PKR-like ER kinase
ATF6: Activating transcription factor 6
BiP: Binding immunoglobulin protein
IRE1β: IRE1 beta
RNase: Endoribonuclease
XBP1: X-box binding protein 1
RTCB: RNA 2',3'-cyclic phosphate and 5'-OH ligase
XBP1s: Spliced XBP1
ERAD: ER-associated degradation
RIDD: Regulated IRE1-dependent decay
ASK1: Apoptosis signal-regulating kinase 1
JNK: C-Jun N-terminal Kinase
TRAF2: Tumor necrosis factor receptor-associated factor 2
NF-κB: Nuclear factor kappa-light-chain-enhancer of activated B cells
IκBα: Inhibitor of NF-kB alpha
IKK: Inhibitor of NF-kB kinase
AP-1: Activator protein 1
EIF2AK3: Eukaryotic translation initiation factor 2 alpha kinase 3
eIF2α: Eukaryotic initiation factor-2 alpha
ATF4: Activating transcription factor 4
CHOP: CCAAT/enhancer binding protein (C/EBP) homologous protein
uORF: Upstream open reading frame
CDS: Coding sequence
RPAP2: RNA polymerase II associated protein 2
GADD34: Growth arrest and DNA damage inducible protein
PP1: Protein phosphatase 1
ROS: Reactive oxygen species
NRF2: Nuclear factor erythroid 2-related factor 2
TXNRD1: Thioredoxin reductase 1
HO-1: Hemeoxygenase-1
MAMs: Mitochondria-associated membranes
InsP3: Inositol-1,4,5-Trisphosphate
InsP3Rs: Inositol-1,4,5-Trisphosphate Receptors
EMCs: ER–Mitochondria Contact Sites
E-Syt1: Extended-Synaptotagmin 1
ATF6α: Activating transcription factor 6 alpha
ATF6β: Activating transcription factor 6 beta
TAD: Transcriptional activation domain
S1P: Site-1 protease
S2P: Site-2 protease
ERp18: Endoplasmic reticulum protein 18 (Thioredoxin domain containing 12)
PDIA5: Protein disulfide isomerase family A member 5
P58IPK: Protein 58 inhibitor of protein kinase (also known as DNAJC3)
PCa: Prostate cancer
BCa: Breast cancer
PDX: Patient-derived xenograft
MMPs: Matrix metalloproteinases
EMT: Epithelial-to-mesenchymal transition
ECM: Extracellular matrix
TSC1: Tuberous sclerosis complex 1
TSC2: Tuberous sclerosis complex 2
PTEN: Phosphatase and tensin homolog
HRAS: Harvey rat sarcoma viral oncogene homolog
BRAF: V-Raf murine sarcoma viral oncogene homolog B1
RET: RET proto-oncogene
PRRs: Pattern recognition receptors
NOXs: NADPH oxidases
MEFs: Mouse embryonic fibroblasts
HIF1α: Hypoxia-inducible factor 1 alpha
GLUT1: Glucose transporter 1
LDHA: Lactate dehydrogenase A
ERO1α: Endoplasmic reticulum oxidoreductin 1 alpha
HBP: Hexosamine biosynthetic pathway
UDP-GlcNAc: Uridine diphosphate N-acetylglucosamine
SERCA: Sarcoplasmic/endoplasmic reticulum calcium ATPase
ISR: Integrated stress response
DAMPs: Damage-associated molecular patterns
HMGB1: High-mobility group box 1
TLR2: Toll-like receptor 2
TLR4: Toll-like receptor 4
DCs: Dendritic cells
NKp46: Natural killer cell p46-related receptor
NK cells: Natural killer cells
ecto-CRT: Externalized calreticulin
RNS: Reactive nitrogen species
TNBC: Triple-negative breast cancer
GCN2: General control nonderepressible 2
MHC-I: Major Histocompatibility Complex Class I
TAP1: Transporter Associated with Antigen Processing 1
NKG2D: Natural Killer Group 2 Member D
MICA: MHC Class I Polypeptide-Related Sequence A
CAR: Chimeric Antigen Receptor
GM-CSF: Granulocyte–Macrophage Colony-Stimulating Factor
CXCL1: CXC-Chemokine Ligand 1
IL-6/IL-8: Interleukin-6 / Interleukin-8
CCL2: CC-Chemokine Ligand 2
CAFs: Cancer-Associated Fibroblasts
MDSCs: Myeloid-Derived Suppressor Cells
HCC: Hepatocellular Carcinoma
PD-L1: Programmed Cell Death Protein 1 Ligand 1
CD8+: Cluster of Differentiation 8 Positive
PDCD1 / PD-1: Programmed Cell Death 1
ICI: Immune Checkpoint inhibition
CTLA-4: Cytotoxic T-Lymphocyte-Associated Protein 4
NSCLC: Non-Small Cell Lung Cancer
TILs: Tumor-Infiltrating Lymphocytes
TAMs: Tumor-Associated Macrophages
BMDMs: Bone Marrow-Derived Macrophages
VEGFA: Vascular Endothelial Growth Factor A
IL4: Interleukin-4
SIRPα: Signal Regulatory Protein Alpha
THBS1: Thrombospondin 1
Arg1: Arginase 1
TLRs: Toll-Like Receptors
PGE2: Prostaglandin E2
CARM1: Coactivator-Associated Arginine Methyltransferase 1
OSCC: Oral Squamous Cell Carcinoma
LN: Lymph Node
GBM: Glioblastoma
MDMs: Monocyte-Derived Macrophages
LXR: Liver X Receptor
LPCAT3: Lysophosphatidylcholine Acyltransferase 3
PSAT1: Phosphoserine Aminotransferase 1
BAT3: HLA-B Associated Transcript 3
TIM-3: T-Cell Immunoglobulin and Mucin Domain-Containing Protein 3
4BPA: 4-Phenylbutyric Acid
4μ8C: IRE1α inhibitor
MC38: Mouse colon adenocarcinoma cell line
α-T-K: Alpha-Tocopherol-containing Nanoemulsion
G9668: IRE1α kinase domain inhibitor
mtDNA: Mitochondrial DNA
STING: Stimulator of interferon genes
AMG-44: PERK inhibitor
IFNγ: Interferon gamma
CIN: Chromosomal instability
STAT3: Signal transducer and activator of transcription 3
KO: Knockout
LLC: Lewis lung carcinoma
HSPCs: Hematopoietic stem and progenitor cells
GSK2606414: PERK inhibitor
CD4 +: Cluster of Differentiation 4 positive
TAGLN2: Transgelin 2
FABP5: Fatty acid-binding protein 5
LAG3: Lymphocyte activation gene 3
B16-OVA: B16 melanoma cells expressing ovalbumin
CRISPR/Cas9: Clustered regularly interspaced short palindromic repeats / CRISPR-associated protein 9
MKC8866: IRE1α inhibitor
dsRNA: Double-stranded RNA
ZBP1: Z-DNA binding protein 1
NLRP3: NOD-, LRR- and pyrin domain-containing protein 3
GSDMD: Gasdermin D
TBX21 (T-bet): T-box transcription factor TBX21
WT: Wild-type
KIRA6: IRE1α inhibitor
IgM: Immunoglobulin M
CLL: Chronic lymphocytic leukemia
PDAC: Pancreatic ductal adenocarcinoma
GSK2656157: PERK inhibitor
B-I09: IRE1α inhibitor
KIRA8: IRE1α inhibitor
HC-5404: PERK inhibitor
NMS-03597812: PERK inhibitor
IFNAR1: Interferon alpha/beta receptor 1
RIPK1: Receptor interacting serine/threonine kinase 1
KIT: KIT receptor tyrosine kinase
